# Faunistic and bibliographical inventory of the Psychodinae moth-flies of North Africa (Diptera, Psychodidae)

**DOI:** 10.3897/zookeys.558.6593

**Published:** 2016-02-01

**Authors:** Hanan Afzan, Boutaïna Belqat

**Affiliations:** 1 Laboratory «Ecology, Biodiversity and Environment», Department of Biology, Faculty of Sciences, University Abdelmalek Essaâdi, Tétouan, Morocco

**Keywords:** Moth-flies, Psychodinae, checklist, Rif, High Atlas, Morocco, Tunisia, Algeria, Egypt, North Africa

## Abstract

All published records for the 49 species of moth flies known from North Africa are reviewed and discussed: Morocco (27 species), Algeria (33 species), Tunisia (18 species) and Egypt (five species). In addition, records of seven species of Psychodinae new to the fauna of Morocco are added, of which three are new mentions for North Africa (Table 1) and one is a new record for Egypt. *Telmatoscopus squamifer* Tonnoir, 1922 is transferred to the genus *Iranotelmatoscopus* Ježek, 1987, **comb. n**. *Satchelliella reghayana* Boumezzough & Vaillant, 1987 is transferred to the genus *Pneumia* Enderlein, 1935, **comb. n**. *Pneumia aberrans* Tonnoir, 1922 is transferred to the subgenus *Logima*.

## Introduction

Within Psychodidae, the Psychodinae form a highly derived subfamily containing the majority of psychodid species diversity. The world fauna of Psychodinae consists at present of approximately 2000 recognized and described species belonging to approximately 100 genera. Their taxonomy is not yet satisfactory; a universally-agreed, stable classification is still lacking for the world fauna, since different generic and tribal concepts are still followed by several authors (Vaillant 1971–1983, 1990; Duckhouse 1987; Wagner and Beuk 2002; Ježek and Van Harten 2005; Kvifte 2011).

Contributions to Psychodinae of Morocco are very fragmented and remain patchy; the first record in this area was by Tonnoir (1920) and the first study was that of Vaillant (1955). A few years later, the same author (Vaillant 1958) published on the Psychodinae in North Africa and their range in Europe. It took almost thirty years for another work on Psychodidae in southern Morocco; the survey in the High Atlas showed the presence of five species (Boumezzough and Vaillant 1986). As part of a national study on the biota of inland waters, Dakki (1997) conducted an initial inventory of Moroccan Psychodidae, in which ten species were listed as Psychodinae. A recent study (Ježek 2004) showed the presence of a new species of Psychodinae in Morocco; and in 2012 Omelková and Ježek described a new species from the High Atlas. For Algeria the study of Psychodinae started with Eaton (1894, 1896) who mentioned records on Algerian Psychodinae; in 1955 Satchell showed the presence of six new species for country. Later, Vaillant described many species from Algeria between 1971 and 1983. The only important Psychodinae reference from Tunisia is provided by Wagner (1987).

Concerning the Egyptian psychodids, Tonnoir (1920, 1922) recorded four species of Psychodinae.

**Figure 1.**
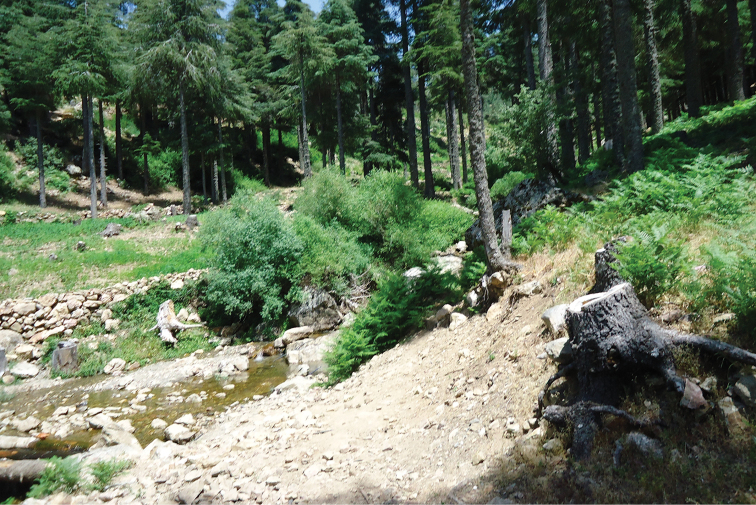
Moroccan habitat of *Pericoma pseudexquisita* Tonnoir, 1940: Oued Azila, mossy and rocky river in cedar forest with dominance of *Pteridium aquilinum* and *Rubus ulmifolius*. Photograph by HA.

**Figure 2.**
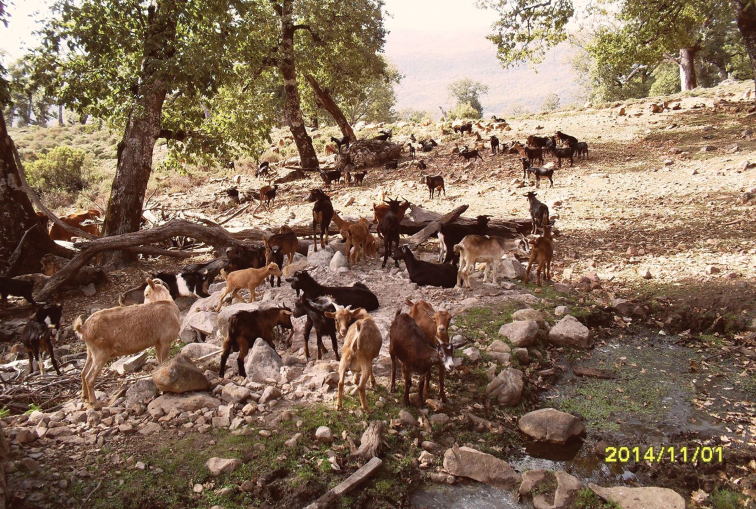
Moroccan habitat of *Pneumia nubila* (Meigen, 1818): Aïn Mâaze, spring with swampy shores, predominant vegetation: *Quercus canariensis*, *Rubus ulmifolius*, *Arbutus unedo*, *Erica arborea*, *Cistus populifolius*, *Luzula* sp. Photograph by HA.

**Figures 3.**
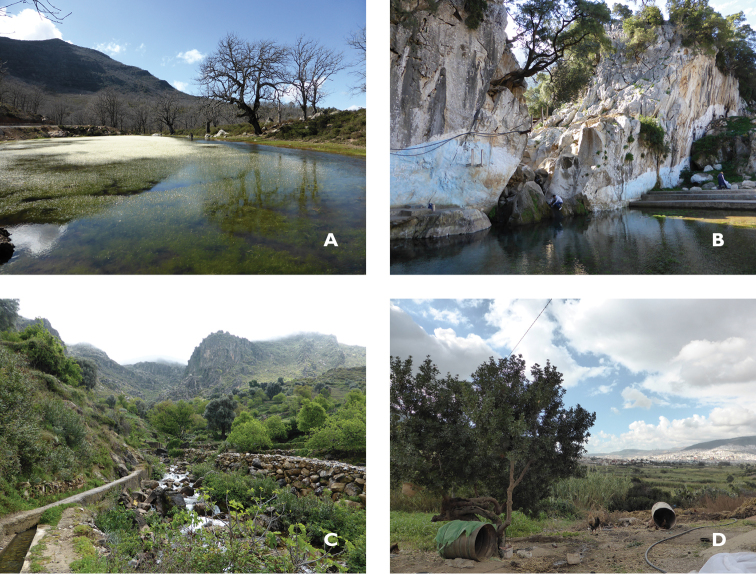
Moroccan habitat of *Psychoda gemina* (Eaton, 1904): **A** Daya Fifi, bog on siliceous sol, predominant vegetation: *Quercus canariensis*, *Quercus pyrenaica*, *Cistus salviifolius*, *Euphorbia characias* **B** Oued Zarka, waterfall and pool with the dominance of moss covering the rocks **C** Oued Aâyaden, river of the high course on a limestone sol with dominance of *Pistachia lentiscus*, *Cistus* sp., *Nerium oleander* and moss on the roc **D** Douar Kitane, farm with *Arondo donax*, *Midicago sativa*, *Inula viscose* and mosses. Photographs by BB.

In our study, a total of 674 specimens (109 larvae, 377 males and 188 females) collected at 47 sampling sites in Morocco and one in Egypt (Table 2) has provided 19 species (18 from Morocco and one from Egypt) and added seven unpublished species to the list of Moroccan Psychodinae: *Clogmia albipunctata* (Williston, 1893), *Psychoda cinerea* Banks, 1894, *Psychoda gemina* (Eaton, 1904), *Pericoma pseudexquisita* Tonnoir 1940, *Philosepedon humerale* (Meigen, 1818), *Pneumia nubila* (Meigen, 1818) and *Pneumia propinqua* (Satchell 1955), and one unpublished species to the Egyptian list: *Psychoda alternata* Say 1824. Of these, *Pericoma pseudexquisita* Tonnoir 1940, *Pneumia nubila* (Meigen, 1818) and *Psychoda gemina* (Eaton, 1904) are first records for North Africa. Locality photos are given in Figures 1, 2 and 3 (A, B, C and D).

**Table 1.** Species (in alphabetical order) of Psychodinae known from the North African countries. Libya has been omitted because no information exists in the literature from Libya.

	Morocco	Algeria	Tunisia	Egypt
*Bazarella atra* (Vaillant, 1955)	X*	X		
*Berdeniella lucasii* (Satchell, 1955)		X		
*Clogmia albipunctata* (Williston, 1893)	X**	X		X
*Clytocerus kabylicus* Wagner, 1987		X		
*Iranotelmatoscopus numidicus* (Satchell, 1955)		X		
*Iranotelmatoscopus squamifer* (Tonnoir, 1922)				X
*Lepiseodina tristis* (Meigen, 1830)		X		
*Mormia tenebricosa* (Vaillant, 1954)	X*	X	X	
*Mormia riparia* (Satchell, 1955)		X		
*Mormia similis* Wagner, 1987			X	
*Panimerus goetghebueri* (Tonnoir, 1919)		X	X	
*Panimerus thienemanni* (Vaillant, 1954)	X	X	X	
*Paramormia ustulata* (Walker, 1856)	X*	X	X	
*Pericoma barbarica* Vaillant, 1955	X*	X	X	
*Pericoma blandula* Eaton, 1893	X	X	X	
*Pericoma diversa* Tonnoir, 1920	X*			
*Pericoma exquisita* Eaton, 1893	X	X	X	
*Pericoma granadica* Vaillant, 1978	X*			
*Pericoma latina* Sarà, 1954	X*	X		
*Pericoma maroccana* Vaillant, 1955	X*			
*Pericoma modesta* Tonnoir, 1922	X	X		
*Pericoma pseudexquisita* Tonnoir, 1940	X***			
*Philosepedon beaucournui* Vaillant, 1974		X	X	
*Philosepedon humerale* (Meigen, 1818)	X**	X		
*Pneumia nubila* (Meigen, 1818)	X***			
*Pneumia pilularia* (Tonnoir, 1940)	X	X		
*Pneumia propinqua* (Satchell, 1955)	X**	X		
*Pneumia reghayana* (Boumezzough & Vaillant, 1986)	X			
*Pneumia toubkalensis* (Omelková & Ježek 2012)	X*			
*Psychoda aberrans* Tonnoir, 1922				X
Psychoda (Falsologima) savaiiensis Edwards, 1928		X		
Psychoda (Logima) albipennis Zetterstedt, 1850		X	X	
Psychoda (Logima) erminea Eaton, 1893		X		
Psychoda (Psycha) grisescens Tonnoir, 1922	X	X	X	
Psychoda (Psychoda) phalaenoides (Linnaeus, 1758)		X		
Psychoda (Psychoda) uniformata Haseman, 1907	X			
Psychoda (Psychodocha) cinerea Banks, 1894	X**	X	X	
Psychoda (Psychodocha) gemina (Eaton, 1904)	X***			
Psychoda (Psychomora) trinodulosa Tonnoir, 1922		X		
Psychoda (Tinearia) alternata Say, 1824	X*	X	X	X**
Psychoda (Tinearia) efflatouni Tonnoir, 1922				X
Psychoda (Tinearia) lativentris Berden, 1952			X	
*Telmatoscopus advena* (Eaton, 1893)		X		
*Thornburghiella quezeli* (Vaillant, 1955)		X	X	
*Tonnoiriella atlantica* (Satchell, 1953)		X	X	
*Tonnoiriella paveli* Ježek, 1999	X			
*Tonnoiriella pulchra* (Eaton, 1893)	X	X		
*Vaillantodes fraudulentus* (Eaton, 1896)		X	X	
*Vaillantodes malickyi* (Wagner, 1987)			X	

X***: new species for North Africa; X**: new species for Morocco or Egypt; X*: new species for the Rif Mountains.

**Table 2.** Sampling sites (in alphabetical order) harboring the species collected in Morocco and Egypt with localities, geographical coordinates and altitudes.

Site	Province, locality	Geographical coordinates	Altitude (m)
Rif Mts			
1. Aïn Bou Ghaba	Chefchaouen, Jbel Bou Bessoui	35°57.980'N/4°43.447'W	1638
2. Aïn Mâaze	Larache, Jbel Bouhachem	35°14.381'N/05°26.316'W	1294
3. Aïn Quanquben	Chefchaouen, Jbel Bou Bessoui	34°57.634'N/4°40.842'W	1596
4. Aïn Sidi Yahya	Berkan, Beni Snassen	34°48.370'N/2°32.408'W	541
5. Âounsar Aheramen	Chefchaouen, Majjou village	35°06.319'N/5°10.820'W	855
6. Cascade Ras El Ma	Chefchaouen, Majjou village	35°6.162'N/5°10.739'W	859
7. Daya Fifi	Chefchaouen, Fifi	35°06.873'N/5°11.338'W	856
8. Douar Derâa	Chefchaouen, Tanakoub	35°10.106'N/5°25.381'W	770
9. Douar Idrene	Chefchaouen, Oued Laou	35°24.942'N/5°12.593'W	460
10. Douar Ihermochene	Chefchaouen, Oued Laou	35°26.602'N/5°11.793'W	405
11. Douar Ikhlafene	Chefchaouen, Oued Laou	35°25.575'N/5°11.807'W	548
12. Douar Kitane	Tétouan, Kitane	35°32.412'N/05°20.393'W	52
13. Douar Mouklata	Tétouan, Mouklata	35°34.551'N/5°21.505'W	9
14. Douar Taria	Chefchaouen, Daradara	35°8.312'N/5°20.991’ W	796
15. Oued Aâyaden	Chefchaouen, Majjou village	35°6.186'N/5°10.935'W	799
16. Oued Achekrade	Tétouan, Douar Aouzighen	35°22.931'N/5°20.364'W	642
17. Oued Ametrasse	Chefchaouen, Chrafate	35°05.014'N/5°5.130'W	828
18. Oued associé à daya Fifi	Chefchaouen, Fifi	35°00.041'N/5°12.166'W	1280
19. Oued Azila	Al hoceima, Jbel Tidghine	34°52.028'N/04°32.609'W	1601
20. Oued à 20 Km de Fifi	Chefchaouen, Fifi	35°02.077'N/5°12.083'W	1020
21. Oued Chrafate	Chefchaouen, Armoutah	35°04.14'N/5°06.66'W	900
22. Oued El Kanar	Chefchaouen, Beni Fenzar	35°10.083'N/5°01.133'W	220
23. Oued El Kanar	Chefchaouen, 2 km de Douar Assoul	35°17.233'N/4°59.639'W	52
24. Oued Farda	Chefchaouen, Akchour,	35°14.350'N/5°10.46'W	420
25. Oued Hachef	Tanger-Azilah	35°31.37'N/05°42.51'W	58
26. Oued Inesmane	Chefchaouen, Adeldal	35°08.595'N/5°05.100'W	1173
27. Oued Jnane en Niche	Jebha, village Jnane en Niche	35°17.040'N/4°51.479'W	46
28. Oued Kelâa	Chefchaouen, Akoumi	35°14.440'N/5°14.542'W	400
29. Oued Madissouka	Chefchaouen, Talassemtane	35°10.622'N/5°08.400'W	1367
30. Oued M’Hannech	Tétouan, Faculty of Sciences	35°33.650'N/5°21.751'W	8
31. Oued Nakhla	Chefchaouen, Koudiet Krikra	35°23.084'N/5°31.448'W	145
32. Oued Ouara	Chefchaouen, Ikadjiouene	35°03.987'N/5°14.005'W	680
33. Oued Ras El Ma	Chefchaouen, Chefchaouen ville	35°10.230'N/5°15.412'W	628
34. Oued Taïda	Larache, Taïda	35°22.099'N/5°32.297'W	494
35. Oued Talembote	Chefchaouen, Talembote	35°15.041'N/5°11.717'W	320
36. Oued Tazzarine	Chefchaouen, Beni Oualal	35°04.347'N/5°19.339'W	242
37. Oued Tiffert	Chefchaouen, Tiffert	35°11.012'N/5°07.573'W	1230
38. Oued Zarka	Tétouan, Zarka	35°31.211'N/5°20.477'W	128
39. Ruisseau Maison forestière	Chefchaouen, Parc National Talassemtane	35°08.076'N/5°08.262'W	1674
40. Seguia barrage Dar Chaoui	Tanger-Azilah, Dar Chaoui	35°31.27'N/05°43.46'W	47
Beni Snassen			
41. Cascade Grotte des Pigeons	Berkan, Beni Snassen	34°49.044'N/5°24.329'W	676
Middle Atlas Mts			
42. Aïn Vittel	Ifrane, Ifrane ville	33°32.87'N/5°6.616'W	1611
43. Gîte Aït Ayoub	Sefrou, Barrage Allal El Fassi	33°55.446'N/4°40.558'W	537
Central Plain (Costal region)			
44. Douar Aoulad Ali	Safi, Jemaâ Shaim	32°20.288'N/8°51.09'W	170
High Atlas Mts			
45. Cascade sur sol cuivreux	Al Haouz, Taddart	31°21.19'N/7°23.54'W	1607
46. Oued Reghaya	Marrakech, Asni	31°14.736'N/7°58.654'W	1189
Egypt			
47. Oued Nile	Nady Tajdif, Giza	30°3.511'N/31°13.013'E	26

## Material and methods

### Collecting

Six techniques have been used to collect Psychodinae: rearing larvae and pupae in the laboratory from collected substrates in the field; adults were collected with sweep net, adhesive papers impregnated with Ricin oil, malaise traps, light traps and aspirators. The early stages of Psychodids were obtained from the lotic and lentic habitats where they may be abundant. Larvae or/and pupae were collected from running (rivers, springs, streams) and standing waters (ponds, lagoons). The substratum was transported to the laboratory and organized on steel gauze net. This is put over a shallow watered dish and left for several days (Wagner 1997).

As the substratum dries out, larvae of Psychodidae fall down into the water and are extracted from the substrate and put into Petri dishes with some rewet substratum from their biotope. The top of the dishes is covered with fine gauze for aeration and the substratum is kept moist by regular water spraying, but not too wet. Larvae are difficult to control, because they bury into the substratum. However, they develop successfully into adults that can be collected by aspiring them from the dishes (pers. obs., approach modified from Wagner 1997). Adults were also collected with hand nets, sweeping through the vegetation preferably at sunset or directly with an aspirator below bridges at daylight. On the other hand, adults were also collected on sticky traps made of paper impregnated with Ricin, placed in different habitats: trees in the field, old urban and animal environments. Whatever the method used, all adult specimens were fixed in 70% ethanol in which they are left until identification. Some species were recognized at 40–80× magnification but for many species, it was necessary to prepare slides, mostly for the close identification of the male genitalia. The method followed was that used by Wagner (1997).

The authors sampled the Moroccan areas from March 2011 to May 2015 and BB captured the Egyptian material in the Nile River in April 2015.

All specimens collected and recorded are deposited in the collection of Diptera in the Laboratory of Ecology, Biodiversity and Environment, Faculty of Sciences, University Abdelmalek Essaâdi, Tétouan.

The following checklist summarizes the species presently known from North Africa. Those species which are new records for North Africa are marked with three asterisks (***), those new for Morocco or Egypt are marked with two asterisks (**) and the species which represent the first record in the Rif Mountains are signalized with one asterisk (*) (Table 1). Taxa are listed according to the classification scheme of Vaillant (1990), Wagner (1990) and Kvifte et al. (2011).

## Results

### Tribe MARUININI Enderlein, 1937

#### Genus *TONNOIRIELLA* Vaillant, 1982

##### *Tonnoiriella paveli* Ježek, 1999

**Literature records.** Morocco: High Atlas, Anti Atlas (Ježek 1999).

**Comment.** Male described by Ježek (1999: 10–12). Species collected by sweeping on the bank of streams (on *Oleander*, *Ficus* and *Pteropsida*), on wet grassy rocky slopes and on sources in semidesert areas. Considered as mountainous species (Ježek 1999: 10–12).

**Biology.** Unknown.

##### *Tonnoiriella pulchra* (Eaton, 1893)

= *Pericoma pulchra* (Eaton, 1893); Vaillant 1955: 223

**Literature records.** Morocco (Wagner 1990); Algeria: Aurès (Vaillant 1955).

**Biology.** Species found in “madicole” habitat whose substrate consists on bare rock or lined by retaining algae by very few mineral particles, and in compact limestone crust “néoformation” (Vaillant 1955).

##### *Tonnoiriella atlantica* (Satchell, 1955)

= *Pericoma atlantica* Satchell, 1955; Satchell 1955

**Literature records.** Algeria: Fort National, El Biar (Satchell 1955), L’Hospice de Veillards, Bône (Satchell 1955); Tunisia: Oued Titria, Ain Sobah (Wagner 1987).

**Comment.** Male and female described by Satchell (1955: 112–113).

**Biology.** Unknown.

### Tribe PARAMORMIINI Enderlein, 1937

#### Genus *CLOGMIA* Enderlein, 1937

##### **Clogmia albipunctata (Williston, 1893)

= *Telmatoscopus meridionalis* (Eaton, 1894); Tonnoir 1920: 128–133.

= *Telmatoscopus albipunctatus* (Williston, 1893)

**Literature records.** Algeria: Boghari (Alger), Rocher Blanc (Tonnoir 1920, Satchell 1955); Egypt: Delta Barrage, Ghezireh (Tonnoir 1920, El-Badry et al. 2014).

**New records.** Morocco, Rif: Douar Kitane, 13/V/2014, 1♂, 2♀♀, 1/V/2015, 1♀, light trap; Douar Mouklata, 12/IV/2005, 5 larvae, suber net; Oued M’Hannech, 12/IV/2005, 3♂♂, 1/V/2015, 5♂♂, 7♀♀, aspirator; Central Plateau (Coastal region): Douar Aoulad Ali, 12/VII/2014, 1♂, sweep net, coll. Afzan and Belqat.

**New site.** Egypt, Oued Nile: 1–9/IV/2015, 3♀♀, Malaise trap, coll. Belqat.

**Comment.** A complete description and full synonymy of *Clogmia albipunctata* (Williston, 1893) can be found in Ibañez-Bernal (2008).

**Biology.** Species reported, in central Europe in kitchens, bathrooms and hospitals (Oboňa and Ježek 2012). Full bionomies can be found in Boumans (2009), Boumans et al. (2009) and Werner (1997). Collected in the present study in synanthropic habitats and on plants such as *Oxalis*.

#### Genus *LEPISEODINA* Enderlein, 1937

##### *Lepiseodina tristis* (Meigen, 1830)

= *Clogmia tristis* (Meigen, 1830)

= *Telmatoscopus tristis* (Meigen, 1830); Vaillant 1972: 53–54

**Literature record.** Algeria (Vaillant 1972).

**Biology.** Species found in wet rot-holes and in an oak branch-end (Withers 1989). Larvae found in rotting wood or hole trees (Oboňa and Ježek 2012).

#### Genus *IRANOTELMATOSCOPUS* Ježek, 1987

##### *Iranotelmatoscopus numidicus* (Satchell, 1955)

= *Telmatoscopus numidicus* Satchell, 1955: 115

= *Panimerus numidicus* (Satchell, 1955); Vaillant 1972: 78

= *Iranotelmatoscopus numidicus* (Satchell, 1955); Ježek 1987: 6–8

**Literature records.** Algeria: Biskra (Satchell 1955), Vaillant (1972).

**Comment.** Original description of the species *Telmatoscopus numidicus* by Satchell (1955: 115–116).

**Biology.** Unknown.

##### *Iranotelmatoscopus squamifer* (Tonnoir, 1922), comb. n.

*Telmatoscopus squamifer* Tonnoir, 1922: 102

**Literature record.** Egypt: Shoubra (Tonnoir 1922).

**Comment.** *Telmatoscopus squamifer* is transferred to *Iranotelmatoscopus* based on the structure of the male genitalia, ascoids and wing venation, as judged from illustrations.

**Biology.** Unknown.

#### Genus *PANIMERUS* Eaton, 1913

##### *Panimerus goetghebueri* (Tonnoir, 1919)

= *Pericoma goetghebueri* Tonnoir, 1919

= Telmatoscopus (Panimerus) goetghebueri (Tonnoir, 1919); Satchell 1955: 119

= *Telmatoscopus goetghebueri* (Tonnoir 1919); Freeman 1950 (synonymy according to Vaillant 1972)

= *Panimerus goetghebueri* (Tonnoir, 1919); Vaillant 1972: 71

**Literature records.** Algeria: Satchell (1955), Bône (Vaillant 1972); Tunisia: Hammam Bourguiba stream, Hammam Bourguiba, Oued Hammam Bourguiba Barbarian, Ain Sobah, Dum Djeddour, Oued Titria (Wagner 1987).

**Comment.** adults described by Vaillant (1972).

**Biology.** Unknown.

##### *Panimerus thienemanni* (Vaillant, 1954)

= *Mormia thienemanni* Vaillant 1954

= *Telmatoscopus thienemanii* (Vaillant, 1954); Vaillant 1955: 85, 200–202

= ? *Panimerus maynei* (Tonnoir, 1919); Vaillant 1972 (placed in synonymy)

= *Panimerus thienemanni* (Vaillant, 1954); Vaillant and Withers 1992 (raised from synonymy)

**Literature records.** Morocco: High Atlas (Boumezzough and Vaillant 1986); Algeria: Djurdjura mountains (Satchell 1955), (Vaillant 1972; Vaillant and Withers 1992); Tunisia: Oued Maden (Wagner 1987).

**Comment.** Adults reared from larvae collected in the foam in Assif Reghaya by Boumezzough and Vaillant (1986: 237); detailed description of the adult given by Vaillant (1972).

The status of *Panimerus maynei* in North Africa is unclear. Vaillant (1954) described *Mormia thienemanni* from Algeria and later synonymized it with *Panimerus maynei* (Vaillant 1972). However, Vaillant and Withers (1992) identified diagnostic differences between the type material of *Panimerus maynei* and *Panimerus thienemanni* and raised the latter species from synonymy. The records of *Panimerus maynei* given by Satchell (1955), Boumezzough and Vaillant (1986) and Wagner (1987) are here assumed to represent *Panimerus thienemanni*, although the material should ideally be revised.

#### Genus *VAILLANTODES* Wagner, 2001

##### *Vaillantodes fraudulentus* (Eaton, 1896)

= *Pericoma* sp. Eaton, 1896

= *Pericoma fraudulenta* Eaton, 1896

= *Xenapates fraudulenta* (Eaton, 1896); Eaton 1904

= *Telmatoscopus fraudulentus* (Eaton, 1896); Satchell 1955:116–118

= *Panimerus fraudulentus* (Eaton, 1896); Vaillant 1972: 79

= *Jungiella fraudulenta* (Eaton, 1896); Wagner 1987: 17–18

= *Vaillantia fraudulentus* (Eaton, 1896); Wagner 1988: 10

**Literature records.** Algeria: Mt. Edough, l’Hospice de Vaillards (Bône), El Biar, Aine Souk, Forêt de Yakourene (Hakowen), Mustaph Superior (Satchell 1955); Tunisia (Wagner 1987).

**Comment.** Male and female described by Satchell (1955).

**Biology.** Unknown.

##### *Vaillantodes malickyi* Wagner, 1987

= *Jungiella malickyi* Wagner, 1987: 18–19

**Literature record.** Tunisia: Hammam Bourguiba (Wagner 1987).

**Comment.** Description of adult by Wagner (1987: 18–19).

**Biology.** Unknown.

#### Genus *PARAMORMIA* Enderlein, 1935

= *Paramormia* Enderlein, 1935: 248

= *Duckhousiella* Vaillant, 1972: 54

##### *Paramormia ustulata (Walker, 1856)

= *Pericoma ustulata* Walker, 1856: 263

= *Telmatoscopus limosus* Vaillant, 1955: 85

= *Duckhousiella ustulata* (Walker, 1856); Vaillant 1972: 58

= *Paramormia ustulata* (Walker, 1856); Wagner 1990: 50

**Literature records.** Morocco: High Atlas (Vaillant 1955, 1972); Algeria: Djurdjura, Aurès, Petite-Kabylie (Satchell 1955, Vaillant 1955, 1972); Tunisia: Hammam Bourguiba stream, Oued Hammam Bourguiba Barbarian, Ain Drahan, Dum Djeddour, reservoir of Kasseb, Oued Maden (Wagner 1987).

**New record.** Morocco, Rif: Seguia barrage Dar Chaoui, 14/II/2013, 4♂♂, reared; Douar Kitane, 14/XI/2013, 2♂♂, adhesive papers, 24/III/2015, 1♂, malaise trap; Oued Jnane en Niche, 19/IV/2013, 4 ♂♂, sweep net, coll. Afzan and Belqat.

**Comment.** Detailed descriptions of adult, pupae and larvae given by Vaillant (1972: 58–59).

**Biology.** Larva and pupa can live in habitats with different levels of salinity in seaweed-heaps or near salt springs. Larvae can be found in rivers, sea shores, thermal springs, in crust of limestone dust, beneath stones, in moss and in moist earth (Vaillant 1971, 1972). Specimens collected by Ježek (1990a) occupy a large variety of habitats like, banks of outflows of ponds, moist pastures, swamps, steams and pools on margins of forest, arms of rivers, in biotopes with *Alnus*, *Salix*, *Populus*, *Aesculus*, *Pinus*, *Fraxinus* and others.

#### Genus *TELMATOSCOPUS* Eaton, 1904

##### *Telmatoscopus advena* (Eaton, 1893)

= *Pericoma advena* Eaton 1893, 1896

= *Telmatoscopus advenus* Vaillant, 1972: 80

= *Panimerus havelkai* (Wagner, 1975); syn. according to Kvifte (2014): 392

= *Telmatoscopus seguyi* (Vaillant, 1990); syn. according to Kvifte (2014): 392

**Literature record.** Algeria: Fort National (Vaillant 1972).

**Comment.** Vaillant (1972) lists a single specimen that was captured and determined as *Pericoma advena*, but the identification must be considered as doubtful. A full synonymy is given in Kvifte (2014).

**Biology.** Species considered as a tree-breeder; found in sycamore with damp (no standing water) rot, approximately 1.5 m above ground, in elm trunk-base, very damp, but no standing water, in ash, birch, hole approximately 1.5 m. above ground, with some standing water (Withers 1989).

### Tribe Mormiini Enderlein, 1937

#### Genus *MORMIA* Enderlein, 1935

##### *Mormia tenebricosa Vaillant, 1954

= *Telmatoscopus tenebricosus* Vaillant, 1955: 85; Vaillant 1974: 135

**Literature records.** Morocco: High Atlas (Vaillant 1955, 1974); Algeria: Aurès, Petite-Kabylie, Alger (Vaillant 1955, 1974); Tunisia: Hammam Bourguiba, Hammam Bourguiba stream, Ain Drahan, Oued Ain Bousabala, Oued Maden (Wagner 1987).

New record: Morocco, Rif: Oued Achekrade, 9/III/2014, 1♂, reared, coll. Afzan and Belqat.

**Comment.** Detailed description of adult, pupae and larvae (Vaillant 1974: 135–139).

**Biology.** In the present work, larvae were collected and reared by the authors at laboratory temperature; the emergence of the adult took 10 days.

##### *Mormia similis* Wagner, 1987

**Literature records.** Tunisia: Oued Hammam Bourguiba Barbarian (Wagner 1987).

**Biology.** Unknown.

##### *Mormia riparia* (Satchell, 1955)

= Telmatoscopus (Mormia) riparius Satchell, 1955: 113–115

= *Mormia riparia* (Satchell, 1955); Vaillant 1975: 144

**Literature records.** Algeria: Bône, El Biar (Satchell 1955), (Vaillant 1975).

**Comment.** Descriptions of the male (Satchell 1955: 113–115).

### Tribe PERICOMAINI Enderlein, 1935

#### Genus *BAZARELLA* Vaillant, 1964

##### *Bazarella atra (Vaillant, 1955)

= *Pericoma atra* (Vaillant, 1955); Vaillant 1983: 337–339

**Literature records.** Morocco, High Atlas: Massif du Siroua (Vaillant 1955); Algeria: Aurès, Tlemcen, Djurjura, Petite-Kabylie, Massif des Aures (Vaillant 1955, 1983).

**New records.** Morocco, Rif: Oued Inesmane, 12/IV/2004, 1 larva, surber net; Oued Madissouka, 18/V/2014, 5♂♂, sweep net; Aïn Quanquben, 28/IV/2015, 3♂♂, 10♀♀, sweep net; Aïn Bou Ghaba, 28/IV/2015, 1♂, 4♀♀, sweep net; Oued Aâyaden, 27/IV/2015, 2♂♂, aspirator; High Atlas: Oued Reghaya, 07/V/2011, 8 larvae, surber net, coll. Afzan and Belqat.

**Comment.** Larvae, pupae and adults described by Vaillant (1983: 337–339). Species wrongly recorded as new for Morocco by Ježek (2004: 146–147).

**Biology.** Authors of this paper collected the material in rivers, springs and brook with cedar forest and *Rubus ulmifolius* as the predominant vegetation.

#### Genus *BERDENIELLA* Vaillant, 1976

##### *Berdeniella lucasii* (Satchell, 1955)

= *Pericoma lucasii* (Satchel, 1955); Satchell 1955: 111–112.

**Literature records.** Algeria: Coastal city Bône (Satchell 1955, Vaillant 1976).

**Comment.** Adult described by Vaillant (1976: 188).

#### Genus *CLYTOCERUS* Eaton, 1904

##### *Clytocerus kabylicus* Wagner, 1987

= *Clytocerus wollastoni* Satchell, 1953; Satchell 1955: 107–109 (partim, misidentification)

**Literature records.** Algeria: El Biar (Wagner 1987: 14).

**Comment.** *Clytocerus wollastoni* Satchell, 1955 was recorded from Algeria by Satchell (1955), but according to Wagner (1987), these specimens were likely misidentified *Clytocerus kabylicus*. True *Clytocerus wollastoni* occurs only on Madeira.

#### Genus *PERICOMA* Walker, 1856

##### *Pericoma barbarica Vaillant, 1955

**Literature records.** Morocco: High Atlas (Vaillant 1955); Algeria: Aurès, Tlemcen, Edge of Tlemcen, Oued Safsaf, Constantine, Petite Kabylie (Vaillant 1955); Tunisia: Hammam Bourguiba stream, Ain Drahan (Wagner 1987: 13).

**New record.** Morocco, Rif: Oued Taïda, 17/IV/2013, 1♂, 1♀, reared; Douar Taria, 08/IX/2013, 4♂♂, adhesive papers; Cascade Grotte des pigeons, 5/XI/2014, 3♂♂, sweep net, coll. Afzan and Belqat.

**Biology.** In the present work, the adults were collected from vegetation as, *Eucalyptus*, *Olea oleaster*, *Rubus ulmifolius*, *Crataegus monogyna*, *Nerium oleander*, *Chamaerops* sp., and *Phragmites australis* by a waterfall. Adults were also reared at the laboratory temperature from larvae collected in a stony ground stream with brown algae and mosses. They emerged in 30 days.

##### *Pericoma blandula* Eaton, 1893

**Literature records.** Morocco: High Atlas (Boumezzough and Vaillant 1986; Ježek 2004), Rif (Ježek 2004); Algeria: Ruisseau des singes (Vaillant 1979); Tunisia: Hammam Bourguiba stream, Ain Drahan, Oued Sardouk, Oued Titria (Wagner 1987; Ježek 2004).

**New site.** Morocco, Rif: Oued Taïda, 17/IV/2013, 2♂♂, reared; Âounsar Aheramen, 10/V/2014, 9♂♂, 6♀♀, reared; Oued Beni Ouachekradi, 24/XI/2014, 2♂♂, 6♀♀, reared, Oued Aâyaden, 27/IV/2015, 6♂♂, aspirator; Cascade Ras El Ma, 27/IV/2015, 2♂♂, sweep net, coll. Afzan and Belqat.

**Comment.** Adults reared from larvae collected along the Assif Reghaya by Boumezzough and Vaillant (1986: 237); adult, larvae and pupa, habitat of different states and characteristics of *Pericoma blandula* of North Africa and Europe were described by Vaillant (1979: 239–240). Species wrongly recorded as new for Morocco by Ježek (2004: 147).

**Biology.** According to Duckhouse (1962) and Vaillant (1976), the larvae of *Pericoma blandula* can live in different habitats: in mosses which cover, the dead leaves present in the banks of springs and rivers, as well as in sand, mud and stones on the edge of large and small courtyards water. They also can be found in different substrates: granite, basalt and slate. Vaillant (1979) described in detail the larva.

##### *Pericoma granadica Vaillant, 1978

**Literature records.** Morocco: High Atlas (Boumezzough and Vaillant 1986).

**New records.** Morocco, Rif: Oued Taïda, 18/III/2011, 2 larvae, surber net; Oued Ametrasse, 16/V/2011, 9 larvae, surber net; Oued Ras El Ma, 17/V/2011, 2 larvae, surber net; Oued Farda, 28/III/2012, 11♂♂, 2♀♀, sweep net, reared; Oued Aâyaden, 27/IV/2015, 13♂♂, sweep net; Middle Atlas: Aïn Vittel, 11/XII/2011, 4♂♂, 5♀♀, reared; High Atlas: Cascade sur sol cuivreux, 06/V/2011, 2 larvae, surber net; Oued Reghaya, 07/V/2011, 2♂♂, 1♀, sweep net, coll. Afzan and Belqat.

**Comment.** Adults reared from larvae collected in the foam in the site Assif Reghaya (Boumezzough and Vaillant 1986: 237–238).

**Biology.** Larvae extremely abundant in the foam that cover the walls of irrigation canals and exterior walls; adults obtained by breeding (Vaillant 1978). In the present paper, the eclosion at the temperature laboratory of several adults was registered at 2 days from pupae and 60 days from larvae. Adults were also collected by sweeping the vegetation mostly constituted by *Nerium oleander*, *Pistacia lentiscus* and *Rubus ulmifolius* near springs, streams and waterfall habitats.

##### *Pericoma exquisita* Eaton, 1893

= *Pericoma minutissima* Vaillant, 1963

= *Pericoma petricola* Vaillant, 1962

**Literature records.** Morocco: High Atlas, Rif (Ježek 2004); Algeria: Ježek (2004); Tunisia: Hammam Bourguiba stream, Oued maden, Oued Titria (Wagner 1987).

**Biology.** Larvae living on the banks of rivers; adults found on Crete and the islands of Evia in the Aegean (Vaillant 1978).

##### *Pericoma diversa Tonnoir, 1920

**Literature record.** Morocco: High Atlas (Vaillant 1978: 229).

**New record.** Morocco, Rif: Cascade Chrafate, 18/III/2015, 2♂♂, 1♀, reared, coll. Afzan and Belqat.

**Comment.** Description of larva, pupa and male by Vaillant (1978: 229).

**Biology.** Present in fast rivers, fit into the foams containing stones, in walls of natural or artificial waterfalls; as well as in bryophytes covering irrigation canals. In England, larvae were found at an altitude that does not exceed 1100 m; in Morocco it was collected at 2000 m (Vaillant 1978) and at 900 m in the present work.

##### *Pericoma latina Sarà, 1954

= *Pericoma numidica* Vaillant, 1955

**Literature records.** Morocco: High Atlas (Vaillant 1955); Algeria: Aurès, Tlemcen mountains (Vaillant 1978).

**New record.** Morocco, Rif: Cascade Chrafate, 18/III/2015, 2♂♂, reared; Oued Majjou, Nord Village Majjou, 19/03/2004, 1 larva; Oued Majjou, Majjou village, 19/03/2004, 17 larvae; Oued Kelâa, 04/V/2004, 29 larvae; Oued Talembote, 21/VI/2005, 4 larvae; Oued associé à daya Fifi, 16/VI/2005, 25 larvae; Oued Tiffert, 16/VI/2005, 3 larvae; Oued à 20 Km de Fifi, 16/VI/2005, 1 larva; Oued El Kanar, Beni Fenzar, 21/VI/2004, 1 larva, surber net, coll. Afzan and Belqat.

**Comment.** Detailed description of larvae, pupae and adults, reared from larvae (Vaillant 1978: 234–235).

**Biology.** Larvae particularly “petrimadicolous”; can be found also under the leaves soaked on the banks of sources. In the present work, the authors collected the larvae in diversified habitats, in streams, in arms of pounds and rivers, in waterfall. The reared adults were obtained at the temperature laboratory from larvae collected in a waterfall which abundant vegetation was: *Olea oleaster*, *Ficus carica*, *Rubus ulmifolius*, *Eucalyptus*, *Nerium oleander*, *Hedera maroccana* and *Ricinus communis*.

##### *Pericoma maroccana Vaillant, 1955

= Pericoma numidica var. marocana Vaillant, 1955

**Literature records.** Morocco: High Atlas (Boumezzough and Vaillant 1986; Dakki 1997).

**New records.** Morocco, Rif: Cascade Chrafate, 18/III/2015, 2♂♂, 2♀♀, sweep net; Ruisseau Maison forestière, 21/IV/2015, 1♂, sweep net, coll. Afzan and Belqat.

**Comment.** Species recorded from Tissaout in the High Atlas; it is endemic from Morocco.

**Biology.** The authors of the present paper collected the species on the branches of the vegetation around a waterfall and a streamlet. The localities with *Olea oleaster*, *Ficus carica*, *Rubus ulmifolius*, *Eucalyptus*, *Nerium oleander*, *Hedera maroccana*, *Ricinus communis*, *Abies marocana*, *Pinus negra*, *Pinus pinaster*, *Cedrus atlantica* and *Berberis hispanica*.

##### *Pericoma modesta* (Tonnoir, 1922)

= *Pericoma numidica* Vai1lant, 1955 (syn. according to Vaillant 1978)

**Literature records.** Morocco: High Atlas: Boumezzough and Vaillant (1986); Algeria: Aurès, Djurdura, Constantine, Atlas de Blida, Ruisseau des singes, Camp-des-Chênes, Sidi-Madani, Alger (Vaillant 1955), Aegean, Djurdjura mountains (Vaillant 1978).

**Comment.** Adults reared from larvae collected in wet sand along the Assif Reghaya (Boumezzough and Vaillant 1986: 237). Detailed description of larvae and adults (Vaillant 1978: 226–227).

**Biology.** Unknown.

##### ***Pericoma pseudexquisita Tonnoir, 1940

= *Pericoma avicularia* Tonnoir, 1940; Vaillant 1978: 233

**New record.** North Africa, Morocco, Rif: Oued Azila, 27.VI.2013, 7♂♂, 2♀♀, reared, coll. Afzan and Belqat.

**Biology.** Larvae living on pure rocky soil, in the foam and between the leaves. Adults observed throughout the summer season (Vaillant 1978). In the present work, adults were reared at the laboratory and the hatchings were obtained at the 10th and the 20th days. At the unique locality, the most abundant vegetation was formed by *Pteridium aquilinum* and *Rubus ulmifolius*, and the rocky substrate was covered by some mosses.

#### Genus *THORNBURGHIELLA* Vaillant, 1982

##### *Thornburghiella quezeli* (Vaillant, 1955)

= *Pericoma quezeli* (Vaillant 1955)

**Literature records.** Algeria: Petite-Kabylie, Camp-des-Chênes, Constantine, Atlas de Blida, Chabet-el-Akra Vaillant (1955); Tunisia: Aïn Draham (Vaillant 1983).

**Comment.** Detailed description of adult, pupa and larvae (Vaillant 1983: 326–328).

**Biology.** Unknown.

#### Genus *PNEUMIA* Enderlein, 1935

= *Satchelliella* Vaillant, 1979

##### ***Pneumia nubila (Meigen, 1818)

= *Satchelliella nubila* (Meigen, 1818); Vaillant 1979: 270

**New record.** Morocco, Rif: Aïn Mâaze, 1/XI/2014, 1♂, sweep net, coll. Afzan and Belqat.

**Biology.** Larvae found in accumulations of dead, leaves decaying on the bottom of a tank near a stream, or on the banks of a marsh (Vaillant 1981). In the present work, the authors collected the unique adult by sweeping the vegetation formed essentially by *Quercus canariensis*, *Rubus ulmifolius*, *Arbutus unedo*, *Erica arborea*, *Cistus populifolius* and *Luzula* sp.

##### *Pneumia pilularia* (Tonnoir, 1940)

= *Pericoma pilularia* Tonnoir, 1940; Satchell 1955: 118

= *Satchelliella pilularia* (Tonnoir, 1940); Vaillant 1981: 277–278

**Literature records.** Morocco (Ježek 2004); Algeria: Djurdjura mountains (Satchell 1955).

**Comment.** Description of larvae and adult (Vaillant 1981: 277–278).

**Biology.** Larvae common among the remaining plants on the banks of rheocrene springs, many madicole habitats and on limestone substrates (Vaillant 1981).

##### **Pneumia propinqua (Satchell, 1955)

= *Pericoma propinqua* Satchell, 1955; Satchell 1955: 109–111

= *Satchelliella propinqua* (Satchell, 1955); Vaillant 1979: 265–266

**Literature records.** Algeria: Village Tissadourt (Satchell 1955), Tissadourt, Algiers, Fort National in Kabylia (Vaillant 1979).

New Record: Morocco, Rif: Chrafate, 24/V/2013, 2♂♂, reared; Oued Zarka, 14/XI/2013, 2♂♂, reared, coll. Afzan and Belqat.

**Comment.** Description of the male (Satchell 1955: 109–111, Vaillant 1979), placement in *Pneumia* according to Omelková and Ježek (2012).

**Biology.** The authors of the present work reared the species at temperature laboratory; the emergence of the adults was registered after 30 days. The abundant vegetation at the localities was: *Olea oleaster*, *Ficus carica*, *Rubus ulmifolius*, *Eucalyptus*, *Nerium oleander*, *Hedera maroccana* and *Ricinus communis*.

##### *Pneumia reghayana* (Boumezzough & Vaillant, 1986), comb. nov.

= *Satchelliella reghayana* Boumezzough & Vaillant, 1986: 238–239; Dakki 1997: 87, 89

**Literature records.** Morocco: High Atlas (Boumezzough and Vaillant 1986, Dakki 1997).

**Comment.** Adults reared from larvae, description, differential diagnosis (Boumezzough and Vaillant 1986: 238–239). The species was overlooked by Omelková and Ježek (2012) in their catalogue of world *Pneumia* species and is here first recognized as a species of *Pneumia*.

**Biology.** Unknown.

##### *Pneumia toubkalensis (Omelková & Ježek, 2012)

**Literature records.** Morocco: High Atlas (Omelková and Ježek 2012).

New record: Morocco, Rif: Oued Aâyaden, 27/IV/2015, 4♂♂, sweep net; Aïn Ras El Ma, 27/IV/2015, 1♂, sweep net, coll. Afzan and Belqat.

**Comment.** *Pneumia toubkalensis* can be separated from *Pneumia reghayana* on the presence of four digitiform sensilla laterosubapically and a microseta mediosubapically on the gonostyle.

**Biology.** The species was collected on a wall of a river of the higher course, on a limestone soil and mosses on the rock, and on a wall of a spring. The localities were dominated by *Pistachia lentiscus*, *Cistus* sp. and *Nerium oleander*.

### Tribe PSYCHODINI Newman, 1834

#### Genus *PHILOSEPEDON* Eaton, 1904

##### **Philosepedon (Philosepedon) humerale (Meigen, 1818)

= *Psychoda humeralis* Meigen, 1818; Eaton 1893, Satchell 1955: 119, Tonnoir 1919, 1922, Enderlein 1937, Freeman 1950, Jung 1956, Vaillant 1960

**Literature record.** Algeria (Satchell 1955).

**New records.** Morocco, Rif: Oued Hachef, 4/II/2013, 2♂♂, 1♀, reared; Cascade Ras El Ma, 27/IV/2015, 1♀, aspirator; Oued El Kanar, 2 km de Douar Assoul, 27/IV/2015, 1♂, aspirator; Oued Aâyaden, 27/IV/2015, 1♂, aspirator, coll. Afzan and Belqat.

**Biology.** Larvae growing in snail-shells; adults found in damp places (Ježek 1985). The authors of the present work collected the adults on walls of a river of the higher course and of a spring. The localities had a dominance of *Pistachia lentiscus*, *Cistus* sp. and *Nerium oleander*.

##### Philosepedon (Philosepedon) beaucournui Vaillant, 1974

**Literature records.** Algeria (Vaillant 1974); Tunisia: Oued Ain Bousabala, reservoir of Kasseb, Ain Drahan (Wagner 1987).

**Comment.** Description of adult from Algeria. Brief comparison between this species and *Philosepedon humerale* (Vaillant 1974: 116–117).

#### Genus *PSYCHODA* Latreille, 1796

##### Subgenus *Falsologima* Ježek and Van harten, 1996

###### Psychoda (Falsologima) savaiiensis (Edwards, 1928)

= *Psychoda rarotongensis* Satchell, 1953: 183–184

**Literature record.** Algeria (Satchell 1955).

##### Subgenus *Logima* Eaton

###### Psychoda (Logima) aberrans Tonnoir, 1922

**Literature record.** Egypt: Shoubra (Tonnoir 1922)

**Comment.** The species is transferred to subgenus *Logima* based on figures in Tonnoir (1922).

**Biology.** Unknown.

###### Psychoda (Logima) albipennis Zetterstedt, 1850

= *Psychoda severini* Tonnoir, 1922; Ježek, 1983: 214

**Literature records.** Algeria (Satchell 1955); Tunisia: Hammam Bourguiba stream, Ain Drahan (Wagner 1987).

**Biology.** Larvae living in various habitats: in the mud of tracks of both cattle and horses, dung, waste pipes drain devices out houses and on the trickling beds of sewage films, bathrooms (Wagner 1977).

###### Psychoda (Logima) erminea Eaton, 1893

**Literature records.** Algeria (Satchell 1955).

**Biology.** Larvae found on the margins of polluted ponds or reservoirs (Nielsen 1961), on banks of streams and drainage canals, swamps, periphery of ponds (Vaillant and Botosaneanu 1966); adults have been collected in localities shaded by *Alnus*, *Salix*, *Robinia*, *Sambucus*, *Pinus* and *Fraxinus*, with undergrowth with mostly *Geranium* and *Urtica* (Ježek 1983).

##### Subgenus *Psycha* Ježek, 1984

###### Psychoda (Psycha) grisescens Tonnoir, 1922

**Literature records.** Morocco: Rif (Ježek 2004); Algeria (Satchell 1955); Tunisia: Hammam Bourguiba stream (Wagner 1987).

**New records.** Morocco, Rif: Douar Kitane, 13/III/2014, 1♂, 3♀♀, sweep net, 20-22/IV/2015, 14♂♂, light trap, 1/V/2015, 4♂♂, light trap, 24/III/2015, 60♂♂, 5♀♀, malaise trap, H. Afzan and B. Belqat collectors; Middle Atlas: Gîte Aït Ayoub, 14/IV/2014, 2♂♂, adhesive papers, coll. Afzan and Belqat.

**Biology.** Larvae found on banks of polluted brooks or in wet cow dung; adults collected in banks of a pond, on house windows, on the branches of coniferous trees and in gardens (Ježek 1990b).

##### Subgenus *Psychoda* s. str.

###### Psychoda (Psychoda) phalaenoides (Linnaeus, 1758)

**Literature record.** Algeria (Satchell 1955).

**Biology.** Adults found in several habitats: banks of mountain forest brooks, decaying organic matter in drainages, growth of alders, dry places, banks of rivers, springs on meadows, outflow from ponds and swamps with *Populus*, *Alnus*, *Picea*, *Fagus*, *Castanea*, the undergrowth with *Urtica*, *Petasites*, *Imoatiens*, *Ficaria*, *Grossularia*, *Ires*, *Rubus*, *Fragaria*, *Filipendula* and *Assarum* (Ježek 1990b).

###### Psychoda (Psychoda) uniformata Haseman, 1907

**Literature record.** Morocco: Rif (Ježek 2004).

**Biology.** Adults found in various habitats: banks of drainages, moist meadows, near arms of rivers, forest brooks pond, in dry bed of canal shaded by *Alnus*, *Fraxinus*, *Crataegus* and others (Ježek 1990b).

##### Subgenus *Psychodocha* Ježek, 1984

###### **Psychoda (Psychodocha) cinerea Banks, 1894

**Literature records.** Algeria (Satchell 1955); Tunisia: Hammam Bourguiba, Hammam Bourguiba stream, Oued Titria, Ain Drahan, Ain Sobah (Wagner 1987).

**New records.** Morocco, Rif: Oued Tazzarine, 17/V/2011, 3♂♂, 7♀♀, sweep net; Douar Taria, 08/IX/2013, 5♂♂, adhesive papers; Douar Kitane, 30/IV/2015, 2♂♂, light trap, 24/III/2015, 4♂♂, malaise trap; Oued Chrafate, 27/IV/2015, 2♂♂, 3♀♀, light trap, 27/IV/2015, 2♂♂, 5♀♀, aspirator, 28/IV/2015, 2♂♂, 2♀♀, sweep net; OuedAâyaden, 27/IV/2015, 2♂♂, sweep net; Beni Snassen: Cascade Grotte des Pigeons, 25/XI/2014, 1♂, reared, coll. Afzan and Belqat.

**Biology.** Larvae registered by several authors (in Ježek 1990b) in diversified habitats (in mud and moss, below stones and moist rock walls, in stagnant waters, in ducts of drainage machinery, on toilets, near banks, in food industry, cow excrements, hollows of trees, heaps of garden’s rest, margins of periodical water reservoirs, etc.). Adults, also collected by several authors (in Ježek 1990b) in light traps, on branches of coniferous trees, in mixed forests, on banks of gutters, brooks, ponds, arms of rivers, in gardens, dirty toilets, etc. Authors of the present work collected the species in several habitats with predominant vegetation as: *Eucalyptus*, *Olea oleaster*, *Rubus ulmifolius*, *Crataegus monogyna*, *Nerium oleander*, *Chamaerops* sp., *Phragmites australis*, *Ficus carica*, *Hedera maroccana* and *Ricinus communis*.

###### ***Psychoda (Psychodocha) gemina (Eaton, 1904)

**New record.** North Africa, Morocco, Rif: Daya Fifi, 30/III/2012, 3♂♂, 2♀♀, sweep net; Oued Zarka, 14/XI/2013, 8♂♂, 1♀ reared; Douar kitane, 20-22/IV/2015, 5♂♂, 1/V/2015, 5♂♂, light trap; Oued Aâyaden, 27/IV/2015, 1♂, aspirator, coll. Afzan and Belqat.

**Biology.** Larvae living in moist mud of paddocks, in manure, in waste pipes, on toilets, sewage work, water mains etc. (Jung 1956), among decayed leaves on the banks of pounds and near springs (Wagner 1977). Ježek (1990b) collected adults near mountain streams drainages, banks of river, inundated lowland forests, surroundings of sluices, moist places near dustbins, rills below railway bridges, spring areas with fallen trees, brooks in meadows, ponds and their outflows, swamps in forests, dry water reservoirs and dry cesspools.

In the present paper, the authors collected the species on both lotic and lentic habitats. The predominant vegetation in the localities were dominated by *Quercus canariensis*, *Quercus pyrenaica*, *Cistus salviifolius*, *Euphorbia characias*, *Arondo donax*, *Midicago sativa*, *Inula viscose* and mosses.

##### Subgenus *Psychomora* Ježek, 1984

###### Psychoda (Psychomora) trinodulosa (Tonnoir, 1992)

**Literature records.** Algeria (Satchell 1955).

**Biology.** Larvae developed in horse and cow excrement (Wagner 1977). Adults collected in areas of inundated forests, on banks of brooks and gutters, on moist pastures, near arms of rivers, rubbish heaps, at moist material, dry drainages, banks of ponds, spring areas and toilets (Ježek 1990b).

##### Subgenus *Tinearia* Schellenberg, 1803

###### *Psychoda (Tinearia) alternata Say, 1824

**Literature records.** Morocco: High Atlas: La Maire (Tonnoir 1920); Algeria (Satchell 1955); Tunisia: Oued Ain Bousabala (Wagner 1987).

New record: Morocco, Rif: Oued Nakhla, 18/III/2011, 7♂♂, 5♀♀, sweep net; Oued Farda, 28/III/2012, 1♀, reared; Oued Ouara, 23/XI/2012, 1♀, reared; Oued Ametrasse, 11/VI/2012, 2♂♂, 4♀♀, reared; Oued Chrafate, 11/VI/2012, 12♂♂, 16♀♀, reared; Douar Derâa, 24/VIII/2013, 2♂♂, 5♀♀, adhesive papers; Douar Ihermochene, 06/V/2014, 9♂♂ 9♀♀, adhesive papers; Douar Ikhlafene, 07/X/2013, 15♂♂, 10♀♀, 06/V/2014, 2♂♂, 6♀♀, adhesive papers; Douar Taria, 08/IX/2013, 4♂♂, 11♀♀, adhesive papers; Douar Idrene, 4♂♂, 2♀♀, 6.X.2013, adhesive papers; Douar Kitane, 9/III/2014, 12♂♂, 20♀♀, light trap; Oued 2km deDouar Assoul, 27/IV/2015, 2♀♀, aspirator; Douar kitane, 1/V/2015, 50♂♂, 6♀♀ light trap; Oued Aâyaden, 27/IV/2015, 1♀, sweep net; Ruisseau Maison forestière, 21/IV/2015, 2♂♂, sweep net; Oued Mhannech, 5♂♂, 7♀♀, aspirator; Aïn Sidi Yahya, 26/XI/2014, 1♂, reared; Middle Atlas: Gîte Aït Ayoub, 14/IV/2014, 1♂, 1♀, adhesive papers, coll. Afzan and Belqat.

**New record.** Egypt, Oued Nile: 3♂♂, 1♀, 1-9/IV/2015, malaise trap, Belqat coll.

**Biology.** The authors of this paper collected the species in varied habitats: rivers, streamlets and walls of homes in small countryside villages (light trap and adhesive papers). The localities whith *Olea oleaster*, *Ficus carica*, *Rubus ulmifolius*, *Eucalyptus*, *Nerium oleander*, *Hedera maroccana*, *Ricinus communis*, *Abies marocana*, *Pinus negra*, *Pinus pinaster*, *Cedrus atlantica*, *Berberis hispanica*, *Pistacia lentiscus* and *Rubus ulmifolius*.

###### Psychoda (Tinearia) efflatouni Tonnoir, 1922

**Literature record.** Egypt: Shoubra (Tonnoir 1922).

**Biology.** Unknown.

###### Psychoda (Tinearia) lativentris (Berdén, 1952)

**Literature record.** Tunisia: Ain Drahan (Wagner 1987).

**Comment.** Cited in Tunisia by Wagner (1987).
